# Usefulness of MRI-based radiomic features for distinguishing Warthin tumor from pleomorphic adenoma: performance assessment using T2-weighted and post-contrast T1-weighted MR images

**DOI:** 10.1016/j.ejro.2022.100429

**Published:** 2022-06-18

**Authors:** Lorenzo Faggioni, Michela Gabelloni, Fabrizio De Vietro, Jessica Frey, Vincenzo Mendola, Diletta Cavallero, Rita Borgheresi, Lorenzo Tumminello, Jorge Shortrede, Riccardo Morganti, Veronica Seccia, Francesca Coppola, Dania Cioni, Emanuele Neri

**Affiliations:** a Academic Radiology, Department of Translational Research, University of Pisa, Via Roma 67, 56126, Pisa, Italy; b Department of Clinical and Experimental Medicine, Section of Statistics, University of Pisa, Via Roma 67, 56126, Pisa, Italy; c Otolaryngology, Audiology, and Phoniatric Operative Unit, Department of Surgical, Medical, Molecular Pathology, and Critical Care Medicine, Azienda Ospedaliero Universitaria Pisana, University of Pisa, 56124 Pisa, Italy; d Department of Radiology, IRCCS Azienda Ospedaliero Universitaria di Bologna, 40138, Bologna, Italy; e Italian Society of Medical and Interventional Radiology, SIRM Foundation, Via della Signora 2, 20122, Milano, Italy

**Keywords:** ADC, apparent diffusion coefficient, AUC, area under the curve, FNAC, fine needle aspiration cytology, GLCM, gray level co-occurrence matrix, GLDM, gray level dependence matrix, GLRLM, gray level run length matrix, GLSZM, gray level size zone matrix, IBSI Image, Biomarker Standardization Initiative, NGTDM, neighboring gray tone difference matrix, PA, pleomorphic adenoma, PcfsT1W, post-contrast fat-suppressed T1-weighted, ROC, receiver operating characteristics, WT, Warthin tumor, Warthin tumor, Pleomorphic adenoma, Head and neck cancer, Parotid neoplasm, Radiomics, Magnetic resonance imaging

## Abstract

**Purpose:**

Differentiating Warthin tumor (WT) from pleomorphic adenoma (PA) is of primary importance due to differences in patient management, treatment and outcome. We sought to evaluate the performance of MRI-based radiomic features in discriminating PA from WT in the preoperative setting.

**Methods:**

We retrospectively evaluated 81 parotid gland lesions (48 PA and 33 WT) on T2-weighted (T2w) images and 52 of them on post-contrast fat-suppressed T1-weighted (pcfsT1w) images. All MRI examinations were carried out on a 1.5-Tesla MRI scanner, and images were segmented manually using the software ITK-SNAP (www.itk-snap.org).

**Results:**

The most discriminative feature on pcfsT1w images was GLCM_InverseVariance, yielding area under the curve (AUC), sensitivity and specificity of 0.9, 86 % and 87 %, respectively. Skewness was the feature extracted from T2w images with the highest specificity (88 %) in discriminating WT from PA.

**Conclusion:**

Radiomic analysis could be an important tool to improve diagnostic accuracy in differentiating PA from WT.

## 1 Introduction

Salivary gland tumors make up approximately 3–6 % of all head-and-neck neoplasms, the majority of them being benign and predominantly affecting the parotid glands. Among them, pleomorphic adenoma (PA) is the most common type, followed by Warthin tumor (WT) [1]. A reliable preoperative differentiation between WT and PA is important for several reasons. In fact, PA can grow , recur easily after surgery, and is associated with a small, but not negligible risk of malignant transformation [1]. In addition, although superficial or partial parotidectomy has long been the most common treatment for WT, in recent years active surveillance has been proposed as an alternative or first choice approach to preserve patients from risk of transient or permanent facial nerve damage, bleeding, and other surgery complications [2], [3].

Clinical signs and symptoms are usually non-specific, with WT usually presenting as a slowly growing, painless mass in people aged over 50 years with a smoking history. Around 15 % of WT are multifocal in the same gland, and approximately 10 % of cases are bilateral [4], [5], [6].

Fine needle aspiration cytology (FNAC) is considered the gold standard for parotid tumor diagnosis, yet it has some limitations (such as tumor location in the deep parotid lobe) and shows highly variable levels of diagnostic accuracy. In this context, variant mucoepidermoid carcinoma (so-called Warthin-like mucoepidermoid carcinoma) may be particularly problematic, highlighting the need for a reliable preoperative differential diagnosis against WT [2].

Regarding preoperative imaging examination, magnetic resonance imaging (MRI) is the preferred imaging modality for patients with a suspected parotid mass, and contrast-enhanced head-and-neck MRI is essential for the initial diagnosis and an accurate assessment of the location and locoregional extension of salivary gland tumors [7]. However, while conventional MRI can provide some diagnostic clues, it is often inconclusive in differentiating benign from malignant parotid gland lesions, and between various types of benign lesions [8], [9].

Radiomics is an emerging approach that allows the extraction of quantitative data from medical images (including MRI) which, correlated with genomic and clinical parameters, are able to improve diagnostic accuracy and aid individualized patient treatment and outcome prediction [10]. Over the last years, some articles have been published trying to differentiate benign from malignant salivary gland tumors, but only some of them have addressed the discrimination of WT from PA, yielding mixed results [11], [12], [13], [14], [15], [16], [17].

Our purpose is to evaluate the role of MRI-based radiomic features in differentiating WT from PA using both T2-weighted (T2w) and post-contrast fat suppressed T1-weighted (pcfsT1w) images.

## 2 Materials and methods

### 2.1 Patient selection and histopathological findings

This was a retrospective study involving 81 patients (44 male, 37 female, median age 56.9 years) with parotid gland lesions, who underwent a head-and-neck MRI examination at our referral center between March 2010 and July 2018. Written informed consent to MRI was obtained from all patients, and institutional review board approval was waived due to the retrospective nature of the study.

All patients were surgically treated at our Institution with partial parotidectomy; histopathological analysis showed 48 PAs and 33 WTs. Pre-operatively, of the 81 patients enrolled, only 52 (29 male, 23 female, median age 58.1 years) underwent MRI with intravenous administration of paramagnetic contrast material, whereas in the remaining 29 patients a noncontrast MRI examination had been performed. Among patients who underwent contrast-enhanced MRI, histopathological analysis revealed that 30 parotid mass were PA and 22 WT.

All MRI examinations were carried out on a commercial 1.5-Tesla whole body MR scanner (Signa HDxt, General Electric, Milwaukee, WI, USA) using a dedicated 16-channel neurovascular coil. The software ITK-SNAP (www.itk-snap.org) was used for manual segmentation of MR images.

### 2.2 Image processing and statistical analysis

One radiologist with 9 years of experience in head-and-neck imaging manually contoured the outer edge of the entire tumor slice by slice, so as to cover the maximum extent of the tumor without exceeding the lesion border in both T2w and pcfsT1w images, where available.

Prior to feature extraction, the N4 bias correction method [18] was applied to source MR images to correct for low frequency inhomogeneity. Images were then normalized, and a quantization of image intensities inside the ROI was done using a fixed number of 60 bins [19], [20], [21]. This intensity discretization method was used according to the Image Biomarker Standardization Initiative (IBSI) recommendation [22].

The dataset was composed of images with different voxel size, so the images were resampled to a uniform voxel size of 0.5 mm × 0.5 mm × 4.8 mm using the BSpline interpolation algorithm. The features assessed in this study were obtained by performing 2D interpolation within the image slice plane, in compliance with the IBSI recommendation in case of slice thickness significantly larger than pixel spacing [22].

For each patient, a total of 105 radiomic features were extracted using the PyRadiomics v3.0.1 software package [23], [24]. Specifically, 7 main classes of radiomic features were considered, including the following: shape-based (14 features), first-order statistics (18 features), gray level co-occurrence matrix (GLCM, 22 features), gray level dependence matrix (GLDM, 14 features), gray level run length matrix (GLRLM, 16 features), gray level size zone matrix (GLSZM, 16 features) and neighboring gray tone difference matrix (NGTDM, 5 features).

The Mann–Whitney test was used to seek which features allowed differentiating PA from WT. During this process, a p-value less than 0.001 was chosen in order to select the most discriminative features, whereas the threshold for statistical significance was set at p < 0.05 for the remaining analysis. Multivariate stepwise logistic regression was applied to determine independent features and eliminate redundant ones. Logistic binary models were considered reliable when they had both sensitivity and specificity of at least 70 %. A receiver operating characteristics (ROC) curve analysis was performed to find cutoff values for the selected features and to determine area under the ROC curve (AUC), sensitivity and specificity values related to those cutoffs.

## 3 Results

### 3.1 Differentiation of PA versus WT on post-contrast T1-weighted images

Among the 105 distinctive features extracted from pcfsT1w images, 15 were statistically significant in the univariate analysis (*p* < 0.0001) in differentiating PA from WT (Table 1).

**Table 1:** Statistically significant radiomic features extracted on pcfsT1w images (univariate analysis, *p* < 0.0001). The most discriminative featureis highlighted in gray.

The most discriminative feature selected using multivariate stepwise logistic regression was *GLCM_InverseVariance.* Based on this model, the following results were obtained: constant = −12.754, B = 40.793 (p = 0.001). The accuracy of the model was 83 %.

**Fig. 1:**
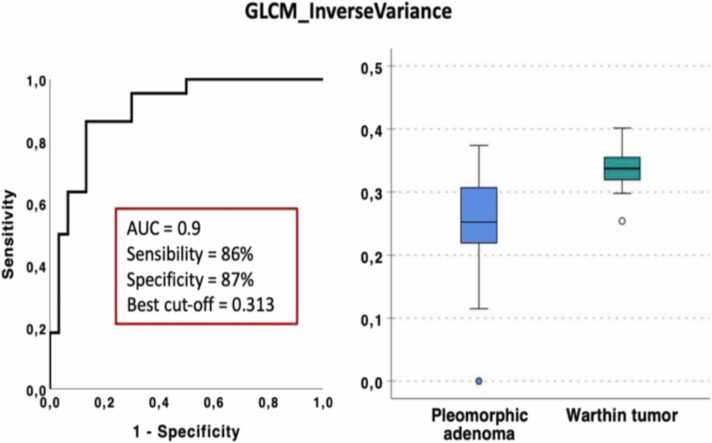
ROC curve analysis and box plot for PA versus WT.

ROC curve analysis (Fig. 1) shows AUC, sensitivity and specificity of 0.9 (95 % CI 0.82–0.98), 86 % and 87 %, respectively.

### 3.2 Differentiation of PA versus WT on T2-weighted images

Several features extracted from T2w images were able to differentiate PA from WT (*p* < 0.0001) (Table 2).

**Table 2:** Statistically significant radiomic features extracted on T2w images (univariate analysis, *p* < 0.0001). The most discriminative features are highlighted in gray.

As a result of the selection process, three features were identified as yielding the most significant discrimination information, i.e., *firstorder_Skewness, GLCM MaximumProbability and GLDM_SmallDependenceHighGrayLevelEmphasis*. Table 3 shows regression coefficients and their statistical significance. The accuracy of the model was 86 %.

**Table 3:** Results of logistic regression analysis.

*Radiomic features*	*B*	*p-value*
FIRSTORDER_Skewness	1.945	0.014
GLCM_MaximumProbability	208.796	0.003
GLDM_SmallDependenceHighGrayLevelEmphasis	–0.004	0.125
Constant	–1.617	0.388

**Fig. 2:**
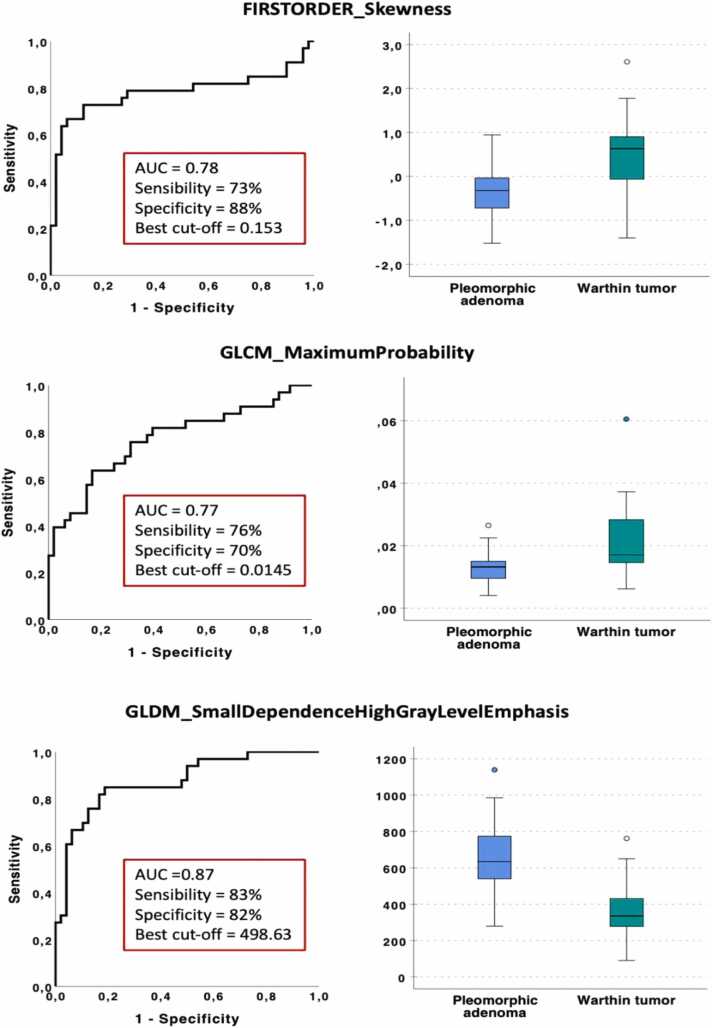
ROC curve analysis and box plots for the three non-redundant features differentiating PA from WT.

The results of the ROC curve analysis are shown for each feature selected in Fig. 2, along with AUC, sensitivity and specificity values. The *GLDM SmallDependenceHighGrayLevelEmphasis* feature obtained the best performance in classifying PA versus WT, with an AUC, sensitivity and specificity of 0.87 (95 % CI 0.79–0.95), 83 % and 82 %, respectively. The highest specificity (88 %, reflecting the ability in correctly identifying WT) was obtained from the *FIRSTORDER_skewness* feature.

## 4 Discussion

Distinguishing WT from PA is of paramount importance due to implications in patient management, treatment and outcome. The accuracy of the diagnosis is based on three steps: clinical data, imaging, and cytology evaluation. When there is discordance regarding WT diagnosis, surgery should be performed to reach the final diagnosis by histopathological examination of the resected specimen [2].

Radiomics is an emerging method that could improve diagnostic accuracy through the quantitative analysis of data from images, and offers advantages over conventional biopsy including noninvasiveness, virtually unlimited repeatability, and possibility to assess the whole tumor tissue and to perform longitudinal follow-up testing [25], [26], [27], [28]. While several articles have been published trying to differentiate benign from malignant salivary gland tumors based on radiomics features extracted from head-and-neck MRI examinations, there is still no consensus on which image sequences should be analyzed, with some studies using T2w images [11] and other ones DWI [15] or a combination of sequences (such as post-contrast T1w, T2w, DCE or apparent diffusion coefficient [ADC] images) [13], [14], [17], [29]. In our study we selected to analyze T2w and pcfsT1w images, because these sequences are an essential part of MRI examinations aimed to the evaluation of a parotid mass, providing key information for the differential diagnosis. Additionally, DWI was not performed in most studies.

As a general concept, radiomics features extracted from T2w images reflect the heterogeneity of the water composition of the tumor, whereas features extracted from pcfsT1w images reflect the heterogeneity of tumor vascularity [30].

Skewness was the feature extracted from T2w images with the highest specificity (88 %) in discriminating WT from PA. Some previous articles [11], [12], [14] reported skewness to allow discriminating WT from PA. In particular, Sarioglu et al. [12] found that WT showed significantly higher skewness on T2w images (*p* < 0.001) than PA, and that skewness was the feature with the largest AUC (0.789; 95 % CI, 0.694–0.885), with a cutoff lower than −0.6895 being associated with PA diagnosis with a sensitivity of 84.4 % and specificity of 62.5 %. Also, Gabelloni et al. [11] found that higher skewness values correlated with the diagnosis of WT, but with lower specificity (78.13 %) and sensitivity (73.91 %). In the current study we found that a higher skewness value (cut-off 0.153) is indicative of WT, possibly related either to the presence of cystic components inside WT or a higher grade of vascularity of WT compared to PA.

Another finding is that the most discriminative feature on pcfsT1w images was GLCM_InverseVariance. Variance is a measure of heterogeneity, with higher values reflecting greater differences in gray level values from their mean. On this basis, a lower GLCM_inverse variance can be associated with higher tissue heterogeneity, in keeping with the literature [31]. Among benign parotid tumors, WT tends to be more heterogeneous than PA [11], so our finding of a higher GLCM_inverse variance in WT would seem contradictory. This finding is in line with those by Piludu et al., who hypothesized that larger lesions had lower dissimilarity and higher energy (hence, higher homogeneity and uniformity) than smaller ones [14], and the WT lesions assessed in our study were larger than PA (9.476 vs 5.570 cm^3^, *p* = 0.001).

Fruehwald-Pallamar et al. [13] and Sarioglu et al. [12] found that texture analysis features derived from pcfsT1w images provided the most significant textural information in the discrimination between PA and WT. Conversely, Vernuccio et al. [29] demonstrated that an MRI-based predictive radiomics model based on texture analysis of T2w images improved the diagnostic performance of non-subspecialized radiologists for the differential diagnosis between PA and WT. Our findings revealed that texture features obtained from T2w and pcfsT1w images can have good discriminatory performance, with skewness derived from T2-weighted images yielding highest specificity.

Our study has some limitations. Firstly, our findings were based on a relatively small patient sample (related to the relatively low prevalence of parotid tumors in the general population), which may have prevented us from obtaining more robust and generalizable data. A second limitation is the lack of external validation using a test set, given the low numerosity of our patient sample.

## 5 Conclusion

Our findings reveal that radiomics analysis of conventional parotid MRI examinations can have a good performance in the preoperative differentiation between WT and PA, with skewness obtained from T2w images showing a specificity as high as 88 %. Further investigations on a larger patient sample with a test set for external validation are warranted to corroborate such findings, which could provide an additional tool for the preoperative diagnostic management of patients with parotid masses.

## Funding sources

This research received no external funding.

## Ethical approval details

Written informed consent to MR imaging for the diagnostic workup of parotid lesions was obtained from all patients and institutional review board approval was waived due to the retrospective nature of the study.

## CRediT authorship contribution statement

Conceptualization, M.G. and L.F.; Formal analysis, R.M., R.B., L.T. and J.S.; Data curation, V.S., F.DV., V.M., J.F. and D.Ca.; Writing – original draft, M.G., F.DV., L.T., R.B and L.F.; writing review and editing, M.G., L.F., F.C., D.C., V.S. and L.T.; supervision, E.N. All authors have read and agreed to the published version of the manuscript.

## Declaration of Competing Interest

The authors declare no conflict of interest.
